# Elimination of Induced Hypoxic Regions in Depth of 3D Porous Silk Scaffolds by the Introduction of Channel Configuration

**DOI:** 10.1155/2022/9767687

**Published:** 2022-03-16

**Authors:** Hadi Tabesh, Zahra Elahi, Zeinab Amoabediny, Fojan Rafiei

**Affiliations:** ^1^Department of Life Science Engineering, Faculty of New Sciences and Technologies, University of Tehran, Tehran, Iran; ^2^Research Center for New Technologies in Life Science Engineering, University of Tehran, Tehran, Iran; ^3^Faculty of Chemical Engineering, University of Tehran, Tehran, Iran

## Abstract

Development of large, clinically sized tissue constructs with efficient mass transport is a tremendous need in tissue engineering. One major challenge in large tissue-engineered constructs is to support homogeneous delivery of oxygen and nutrients throughout the tissue scaffold while eliminating induced hypoxic regions in depth. To address this goal, we introduced an especial channeled architecture on porous silk-based tissue scaffolds to improve supplying of oxygen to the cells in central regions of the scaffolds. Oxygen gradients were measured and evaluated in three scaffold prototypes, namely, one unchanneled and two channeled scaffolds with different channel diameters (500 *μ*m and 1000 *μ*m). The channels were introduced into the constructs using stainless-steel rods arranged uniformly in stainless-steel mold, a fabrication method that enables precise control over channel diameter and the distance between channels. During 2-week culture of G292 cells, the 1000 *μ*m channeled scaffolds demonstrated higher oxygen concentration at the center compared to 500 *μ*m channeled prototype; however, the oxygen concentration approached the same level around the last days of culture. Nevertheless, homogenous oxygen distribution throughout the 1000 *μ*m channeled constructs and the consequence of higher cell proliferation at day 14 postseeding corroborate the efficient elimination of induced hypoxic regions; and therefore, it holds promise for clinically relevant sized scaffold especially in bone tissue engineering.

## 1. Introduction

Tissue engineering is an ideal approach for the regeneration of damaged tissues and also for the fabrication of artificial tissues to study biological functions in vitro [1] or even to be used as a replacement for animal models [2]. In case of bone tissue engineering, the clinical practice for treatment of bone injuries and pathological disease imposes a considerable cost on healthcare system. Over 2.2 million bone-grafting operations take place each year, costing $30 billion worldwide [3, 4]. Considering the increasing demand for bone graft alongside the shortage of supply requires an alternative approach to promote unlimited supply of bone tissue substitute [5]. Scaffold-based bone tissue engineering is a promising alternative that uses cells and bioactive factors on 3D scaffold structures to regenerate damaged bone tissues, caused by, e.g., tumor, trauma, osteoarthritis, and osteonecrosis [6–8].

Considering the limited supply of autografts, immune response risk of allografts, and insufficient osteogenic capacity of bone substitutes, appropriate bioactive bone graft is crucially vital which still remains a major challenge in biomedical engineering [8–11]. An ideal scaffold for bone tissue engineering should mimic osteoblast extracellular matrix (ECM) in order to prepare an environment for cell growth and support the bone remodeling process. These scaffolds are basically fabricated from hyaluronic acid-based hydrogels, heparin-based hydrogels, fibrin-based hydrogels, chondroitin sulfate, or natural polymeric hydrogels [12–14].

Biomaterials such as injectable hydrogels and porous 3D constructs have gained attention due their similarity to ECM and promoting tissue regeneration [15–18].

Silk fibroin is a biocompatible biomaterial which degrades slowly, can be chemically modified, and can be shaped and molded into various structures. The proper physical characteristics of silk fibroin, e.g. strength, light weight, elasticity, and thermal stability, make it a promising biomaterial for tissue engineering [19, 20]. As a result of these inspiring properties, especially its mechanical strength alongside with its highly porous structure, silk could be considered as a suitable biomaterial for fabrication of scaffolds intended to be used in bone tissue engineering [21, 22]. One of the major challenges of silk biomaterial is its poor cell adhesion property which can be solved either using an adjacent natural biomaterial, e.g., gelatin, chitosan, or hyaluronic acid [23–27], or establishing special topographic feature and design configuration [28].

Furthermore, a crucial challenge in engineering sizeable tissue constructs (thickness > 100 − 200 *μ*m) *in vitro* is sufficient supply and homogenous distribution of nutrients and oxygen within the depth of scaffold [29]. In order to bridge this challenge, several approaches have been already proposed to achieve higher oxygen delivery to the cells inside 3D scaffolds including creating convective mass transfer using various bioreactor systems [30, 31], introducing synthetic oxygen carriers to the culture system [32, 33], using oxygen generating biomaterials in the medium [34], developing vessel-like networks in the construct [35], and modifying geometrical design of 3D scaffolds to improve the flow of culture media [36]. Among those aforementioned strategies, an effective approach is to employ methods in order to mimic the native structure of the tissue vascular system [37, 38]. With this respect, the design of scaffold plays a pivotal role since its structure could effectively influence mass transfer, i.e., oxygen and nutrient delivery in depth of scaffold. On the one hand, the microstructure features, e.g., pore size [39], pore interconnectivity [40], and microarchitecture of the pores [41] are quite important. On the other hand, the macrostructure of the scaffold significantly affects the mass transfer inside the construct. In this regard, introducing channels in the structure of tissue constructs attracted a lot of attention among researches in different fields of tissue engineering since channeled scaffold would simulate the capillary network of natural vascularized tissue [42]. Enhanced oxygen delivery through channeled constructs has been demonstrated in previous studies [38, 43].

Various methods have been employed for the fabrication of channeled constructs such as soluble fibers [44], 3D sacrificial molding [42], 3D bioprinting [45], rod removal [46], and soft-lithographic procedure [47, 48] in addition to CO_2_ laser cutting [49], spraying solution onto mold [50], and water jet templating approach [51]. It is apparent that rod removal is a rapid and straightforward as well as a reproducible scaffold fabrication method enabling to precise control over channels' dimension and distribution pattern. For instance, Zhang et al. [52] fabricated channeled scaffolds using linear steel rods and reported improved mass transport. However, a critical question is what would be the appropriate configuration of channels in a 3D clinically relevant sized tissue constructs which could properly eliminate induced hypoxic regions in the depth of scaffolds.

The objective of the present study is to investigate how channel configuration could affect oxygen delivery inside the 3D porous scaffolds. The channeled silk fibroin constructs were fabricated using a set of stainless-steel rods arranged in a well-defined pattern in two groups with channels' diameters of 1000 *μ*m and 500 *μ*m. To ensure the presence of open and interconnected pores with desirable pore size, SEM imaging of the microstructure of scaffolds was performed. Human G292 osteoblast-like osteosarcoma cell lines were cultured and seeded on scaffolds for a period of two weeks. Oxygen concentrations were measured using O_2_ microsensor during culture days at three different points of channeled constructs and compared with unchanneled prototypes. Metabolic activity and viability of G292 cells were examined using Alamar Blue and MTT assays, respectively. In comparison with unchanneled constructs, the results revealed that induced hypoxic regions in depth of our channeled scaffold were efficiently eliminated and cells were attached, grown, and proliferated remarkably through 3D structure. Therefore, this type of channeled 3D scaffold could fulfil variety of needs in engineering of clinically relevant sized tissue constructs especially in bone tissue engineering.

## 2. Materials and Methods

### 2.1. Preparation of Silk Fibroin Solution

Fibroin protein was extracted from silkworm cocoons, a courtesy from Iran Silkworm Rearing Co. (Parnian Branch, Iran), according to the protocol described elsewhere by Rockwood et al. [19], with a minor modification. Briefly, 2.5 g of dried cocoons was boiled in 1 L of 0.02 M Na_2_CO_3_ solution (Merck KGaA, Germany) for 30 min and rinsed 4 times with deionized water to remove sericin proteins completely. The isolated dried fibroin was dissolved in 9.3 M LiBr (Merck KGaA, Germany) aqueous solution, containing 16.14 g LiBr, for 5 h, and subsequently dialyzed against deionized water using dialysis membrane with molecular weight 12000 Da (Sigma-Aldrich Solutions, Germany). The final solution was centrifuged for 30 min at 8500 rpm.

### 2.2. Fabrication of 3D Silk Fibroin Scaffolds

**Figure 1:**
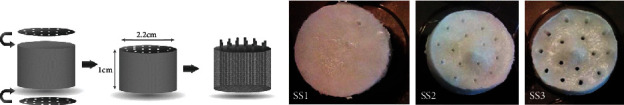
Silk scaffolds. (a) The schematic process of fabricating scaffold prototypes using stainless-steel molds and the top and bottom PMMA sheets as holders of stainless-steel rods. (b) Three groups of fabricated scaffolds after removal of the rods.

Silk scaffolds were fabricated using a modified procedure reported by Rnjak-Kovacina et al. [43]. A key step in the process was to design a mold that enables the fabrication of scaffolds with precise channel diameter at specific positions, which is schematically illustrated in Figure 1(a). With this respect, 15 circular holes distributed evenly, with two different diameters of 500 *μ*m and 1000 *μ*m, were drilled into top and bottom sheets of polymethyl methacrylate (PMMA) (J Lian J Co., Iran) according to the pattern shown in Figure 1(a). These sheets were fixed to the top and bottom of stainless-steel (Mahfanavar Co., Iran) cylindrical molds with inner diameter of 2.2 cm and height of 1.2 cm. Afterwards, linear stainless-steel rods (Mahfanavar Co., Iran), according to the channels diameter with height of 1.5 cm, were arranged into the holes of PMMA sheets and secured by silicon glue (Henkel AG & Co. KGaA, Germany). At the center of top sheets, an extra hole was drilled to facilitate the fabrication procedure of scaffolds. Afterwards, the prepared silk solution was poured into the molds containing stainless-steel rods, through the hole in the center of the top sheet. Samples were then frozen and lyophilized using Dorsatech Laboratory Freeze Dryer (Dorsatech Co., Iran). Subsequently, stainless-steel rods were removed from scaffolds, and the fabricated scaffolds were cut into 1 cm height using a surgical blade (SMI AG Co., Belgium). Finally, the rods were inserted again to get the scaffold prepare for cell seeding procedure.

The scaffold groups SS2 and SS3 are comprised of scaffolds with channel diameters of 500 *μ*m and 1000 *μ*m, respectively, while group SS1 contains no channel. The characteristics of these silk scaffold prototypes are represented in Table 1.

The final three groups of fabricated silk scaffolds are depicted in Figure 1(b) when the stainless-steel rods are removed. Scaffold prototypes were then cross-linked by genipin (Sigma-Aldrich Solutions, Germany) and sterilized by 18 L Multicontrol 2 autoclave (CertoClav Sterilizer GmbH, Austria) prior to cell seeding procedure.

### 2.3. Cell Culture and Cell Seeding

Human G292 osteoblast-like osteosarcoma cell lines (National Cell Bank of Iran, Iran) were expanded in Gibco RPMI 1640 growth medium (Fisher Scientific GmbH, Germany) supplemented with 10% Gibco fetal bovine serum and 1% Gibco penicillin-streptomycin in tissue culture flasks at 37°C with 5% CO_2_ using INC246 CO_2_ incubator (Memmert GmbH + Co. KG, Germany).

The cells were grown and passaged until about 80% confluency and then detached from tissue culture flasks using trypsin-EDTA, 0.25% trypsin with 1 mM EDTA (Sigma-Aldrich Solutions, Germany). Finally, the cells were seeded onto the scaffolds at passages 4-5, i.e., the number of times the culture has been subcultured, harvested, and reseeded into multiple cell culture flasks. Each scaffold prototype was then placed in wells of 6-well plates. For each sample, a total number of 4 × 10^6^ cells were suspended in 0.5 ml medium and seeded on each silk scaffold within four steps every 20 min. The wells were filled with culture medium to cover the top surface of the scaffolds. In order to prevent occupation of channels by cells, 48 h after cell seeding, the stainless-steel rods were removed. 3D cell culture was carried out for 14 days inside INC246 CO_2_ incubator, and media were changed completely every 2 days.

### 2.4. Oxygen Measurement

**Figure 2:**
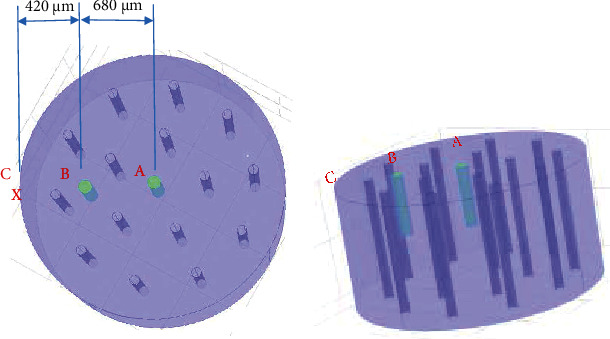
The pattern of arranged 15 channels in the scaffold's structure and oxygen sensing points (A, B, and C). (a) Top view and (b) side view.

NTH-PSt1 oxygen microsensor with its relevant data transmitter Microx TX3 (PreSens Precision Sensing GmbH, Germany) was employed to measure the oxygen at different parts of fabricated silk scaffolds. The oxygen microsensor was calibrated according to manufacturer's protocol before each use. The microsensor needle with 50 *μ*m diameter was sterilized by ethylene oxide gas prior to each investigation. Oxygen concentration was measured in depth of 0.5 cm at three distinct points of each scaffold as shown in Figure 2, namely, point A (at the center), point B (680 *μ*m apart from center), and point C (at the surface). For this purpose, 24 hours after media change (i.e., days 3, 5, 7, and 9), the microsensor needle was inserted on every oxygen sensing points. In order to prevent the microsensor needle from slight quivering, it was mounted on a special holder Test Stand FS-1001 (Lutron Electronic Enterprise Ltd., Taiwan). Oxygen concentration measurements were recorded and sent to a computer using the relevant data transmitter.

Since the microsensor diameter is 50 *μ*m, in order to minimize the possible errors during measurements of next days, after measuring the oxygen concentration in day 3, sterilized stainless-steel rods of diameter 50 *μ*m were used to seal the hole made by oxygen microsensor at positions A and B (refer to Figure 2). The rods were removed and inserted intermittently for oxygen measurements at days 5, 7, and 9.

### 2.5. SEM Observation

Prototypes were investigated by KYKY EM3200 scanning electron microscope (KYKY Technology Development Ltd., China) to study the morphology of scaffolds and cells. Scaffolds were washed with Gibco PBS (Fisher Scientific GmbH, Germany) at day 14 of cell culture and fixed with %1.5 glutaraldehyde (Sigma-Aldrich Solutions, Germany) solution followed by dehydration using gradient ethanol (Sigma-Aldrich Solutions, Germany). To visualize the internal regions, scaffolds were fractured in liquid nitrogen (Arian Gas Co., Iran), sputter coated with gold, and examined by the SEM eventually. To analyze the pore structure of scaffold prototypes, ImageJ software (NIH Image, Bethesda, MD, USA) was utilized.

### 2.6. Cell Proliferation Assay

In order to evaluate cell growth, direct MTT assay was used. The cell-seeded scaffolds were washed twice with PBS and then transferred to the plates. Cell-free scaffolds were used as the control groups. On each scaffold, 1 ml of PBS and MTT solution at a concentration of 5 mg/ml was added. Afterwards, the plates were transferred to the INC246 CO_2_ incubator for 3-4 hours. Then, the scaffolds were taken out from the incubator and the paint was gently removed. To do so, 1 ml of DMSO was added to each scaffold followed by placing in KS 3000i shaker incubator (IKA-Werke GmbH & Co. KG, Germany) at 37°C for 50 minutes at 50 rpm rotation. Then, 100 *μ*l of solution was used to measure the absorption of blue dye at the wavelength of 570 nm with the ELISA reader M965 (Metertech Inc., USA).

To study the effect of the presence of channels as well as channel diameter on cell proliferation, Alamar Blue assay was carried out. At days 7 and 14 of cell culture, the scaffolds were washed twice with PBS and cut into 4 equal pieces. One piece of each scaffold was transferred to a 12-well plate. 1 ml of Alamar Blue solution (MyBioSource Inc., USA) in cell culture medium was added to each sample followed by 4 h shaking incubation using INC246 CO_2_ incubator. Afterwards, 100 *μ*l of Alamar Blue solution from each sample was transferred into a 96-well plate in the absence of light. Fluorescence intensity observation was done at excitation wavelength of 560 nm and emission wavelength of 600 nm using FLx800™ Multidetection Microplate Reader (BioTek Instruments Inc., USA). Unseeded scaffolds were used as controls.

### 2.7. Cell Distribution Assessment

Due to the limited access to confocal microscopy, the staining method was used to examine the penetration and distribution of the cells into the deep layers of the scaffolds. The propidium iodide (PI) (Sigma-Aldrich Solutions, Germany) was used for cell staining. The scaffolds were sectioned into 20 *μ*m thickness by cryocutting knife (Diatome AG, Switzerland). Prior to cutting the scaffolds, the cells were fixed using OCT® adhesive (Fisher Scientific GmbH, Germany).

After washing with PBS, the scaffolds were cut and sectioned using sterilized surgical blades. Subsequently, they were put in aluminum molds filled with OCT® adhesive and transferred to a freezer at -70°C. At this temperature, the cells were fixed inside the scaffolds. Afterwards, very thin layers of scaffold were prepared using cryocutting knife at -30°C; and afterwards, they were placed on a silane slide glass (Corning Life Sciences, USA). At that time, 1 *μ*l of PI dye was poured on each sample; and then, the samples were examined by Nikon ECLIPSE Ti2 fluorescent microscope (Nikon, Japan) and photographed in lightless environment.

### 2.8. Statistical Analysis

Every experiment was conducted at least 3 times, and data was analyzed by SPSS software version 21.0 (IBM SPSS Statistics, USA). Significant differences were calculated using *t*-test assuming unequal variances. A value of *p* < 0.05 was accepted for being statistically significant.

## 3. Results

### 3.1. Scaffold Characteristics

**Figure 3:**
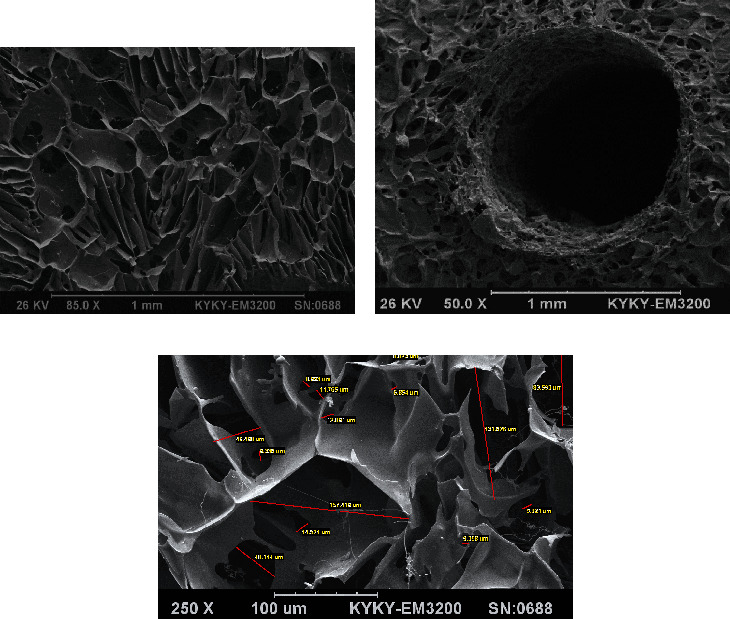
SEM images of SS3 silk scaffold. (a) The surface of scaffold with 85x magnification, (b) a channel with 50x magnification, and (c) ImageJ analysis of pore sizes.

The scanning electron microscopy of silk scaffolds at surface and internal areas was conducted to ensure a uniform hollow structure of channels and analyze the pore characteristics.  Figure 3 demonstrates SEM images of SS3 scaffold prototypes and its channel. Moreover, an analysis of pore diameters, conducted by ImageJ software, is shown in Figure 3(c).

It can be seen in Figure 3(c) that the pore diameter of silk scaffolds ranges from 100 to 300 *μ*m before cell seeding. Pores are distributed throughout the scaffold and are finely interconnected.

### 3.2. Cell Seeding

**Figure 4:**
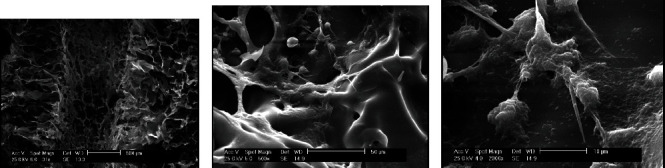
SEM images of SS2 silk scaffold seeded by G292 osteoblastic cells at day 14 with magnification rates of 31x, 500x, and 2000x for (a), (b), and (c), respectively.

After cell seeding, SEM imaging was performed again in order to study the cell attachment on silk scaffolds. Moreover, the morphology of the cells, the cytoskeleton reorganization, and formation of filopodia were thoroughly investigated.  Figure 4 shows SEM images of a cell-seeded SS2 scaffold at day 14 of culture at different magnification rates.

### 3.3. Oxygen Measurement

To compare oxygen profiles in cell-seeded scaffolds in static condition, we monitored oxygen concentrations in the constructs using the very fine and precise oxygen microsensor Microx TX3. The oxygen concentration was measured in the depth of 0.5 cm of scaffolds at three particular positions demonstrated in Figure 2.

**Figure 5:**
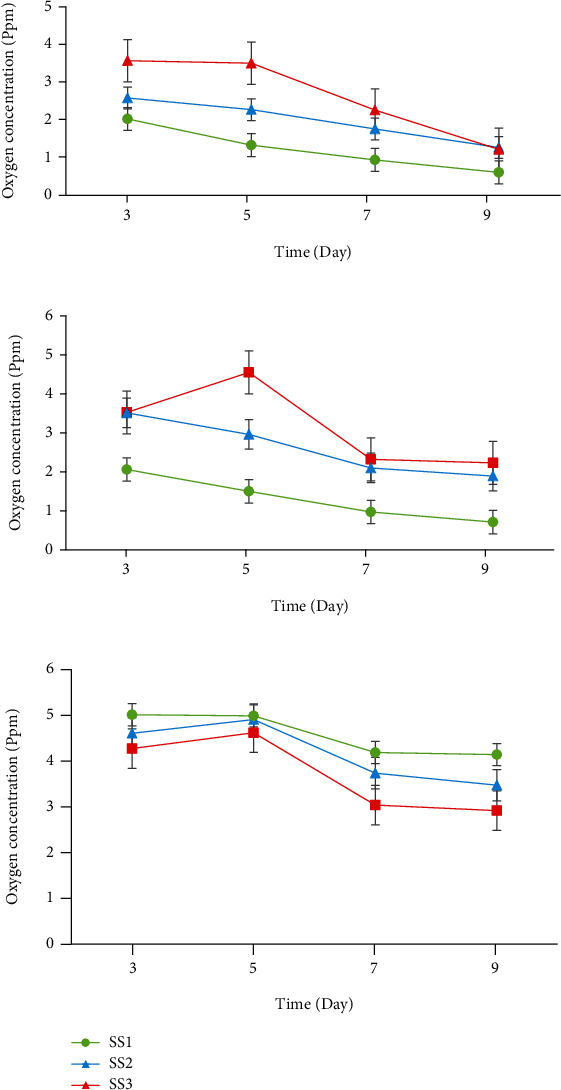
Comparison of oxygen concentration variations between different scaffold prototypes during 9 days in static culture. (a) Point A, (b) point B, and (c) point C.

Figure 5 compares the measured oxygen concentration profiles at days 3, 5, 7, and 9 of culture at points A, B, and C of silk scaffold prototypes (SS1, SS2, and SS3). For each prototype, 5 scaffolds were selected and the oxygen concentrations were carefully measured. Every experiment was repeated 3 times, and the data shown in Figure 5 represents the mean value of measured oxygen concentrations with first standard deviations.

Point A, at the center of scaffold, represents the area of cell residency that has limited accessibility to oxygen and nutrients in culture media, while point C is located at the scaffolds' surface where oxygen and nutrient delivery to cells takes place straightforwardly. Nevertheless, point B, located between A and C, characterizes the oxygen profile through the internal spaces of scaffold prototypes.

### 3.4. Cell Viability

**Figure 6:**
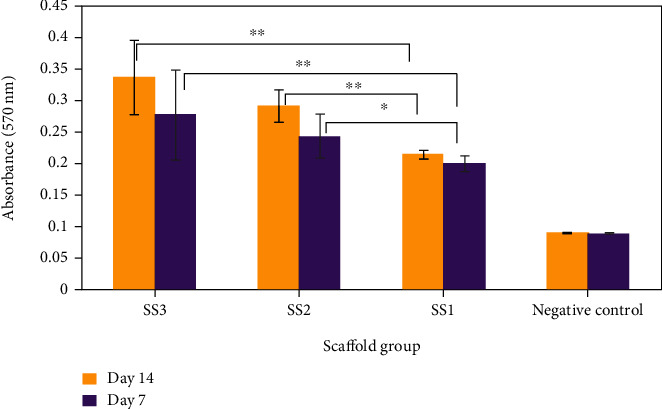
MTT results of G292 cell culture for different groups of scaffolds measured at culture days 7 and 14 (^∗^*p* value < 0.1 and ^∗∗^*p* value < 0.05).

Direct MTT assay was conducted to investigate cell viability on all scaffold prototype groups in comparison with a negative control group.  Figure 6 illustrates the absorbance results of 570 nm wavelength measured by ELISA reader.

**Figure 7:**
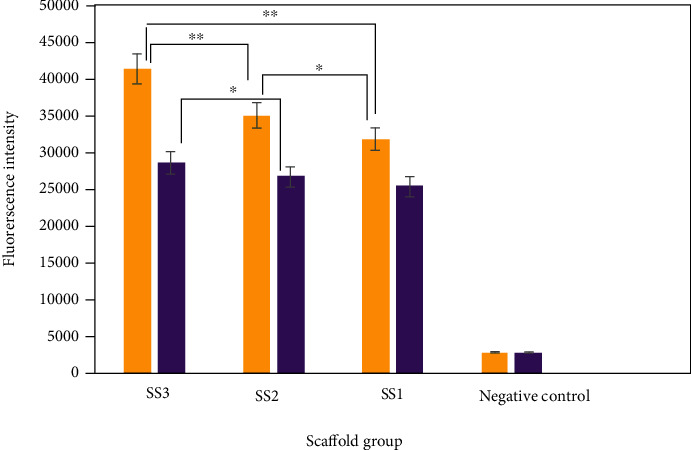
Diagram of G292 metabolic activities for different groups of scaffolds measured at culture days 7 and 14 (^∗^*p* value < 0.1 and ^∗∗^*p* value < 0.05).

To evaluate cell metabolism and proliferation on the 3D cell-seeded silk scaffolds of different prototypes, we performed Alamar Blue assay for periods of 7 and 14 days.  Figure 7 depicts the fluorescent intensities of cell activities at 3 types of silk scaffolds alongside with a negative control.

Observation over a 14-day period cell culture in both Figures 6 and 7 shows a higher cell proliferation on the SS3 group compared to the SS2 group. In addition, lower accessibility to oxygen for SS1 prototypes resulted in a lower cell growth in comparison with the other scaffold groups.

### 3.5. Cell Distribution

**Figure 8:**
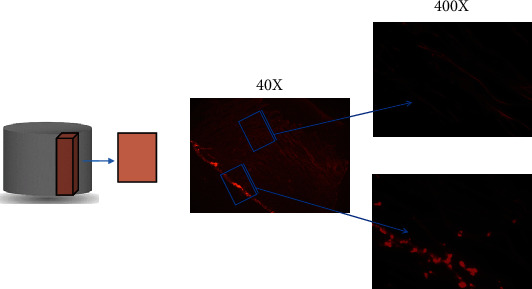
Cell distribution profile on SS1 scaffold. (a) Graphical illustration of SS3 and its vertical cross-section. (b) Captured images under fluorescent microscope with magnification 40x. (c and d) Captured images under fluorescent microscope with magnification 200x at the center and surface of the vertical cross-section, respectively (bright red dots represent G292 osteoblastic cells).

**Figure 9:**
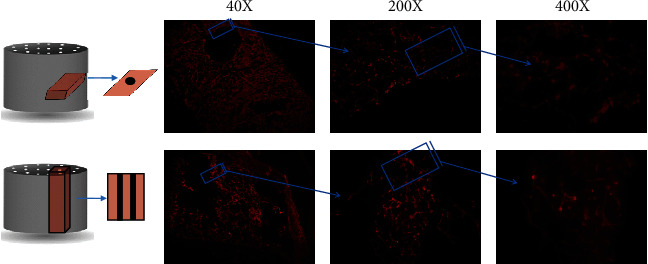
Cell distribution profile on SS3 scaffold. (a) Graphical illustration of SS3 and its horizontal cross-section. (b, c, and d) Captured images under fluorescent microscope with magnifications 40x, 200x, and 400x, respectively. (e) Graphical illustration of SS3 and its vertical cross-section. (f, g, and h) Captured images under fluorescent microscope with magnifications 40x, 200x, and 400x, respectively (bright red dots represent G292 osteoblastic cells).

In order to investigate the effect of channels on cell distribution pattern, PI staining method was performed. Using this method, we investigated the presence of cells in different parts of scaffolds and how the cells were spread throughout channeled and unchanneled prototypes. In this regard, we selected the SS1 and SS3 groups to better visualize the discrepancies between their cell distribution profiles. Vertical and horizontal cross-sections of these scaffolds were cryocut and examined under Nikon ECLIPSE Ti2 fluorescent microscope. Captured images of G292 osteoblastic cell distribution pattern on SS1 and SS3 scaffolds are demonstrated in Figures 8 and 9, respectively, at different magnification ratios.

## 4. Discussion

Generally speaking, the oxygen diffusion from medium through the scaffold is limited to 100-200 *μ*m; and therefore, interior areas of 3D thick constructs are prone to hypoxia [5]. In the present study, the specific pattern of channel's arrangement through the scaffold structure, shown in Figure 2(b), was designed in order to facilitate uniform oxygen distribution through silk scaffolds. In addition to macroscopic configuration, the microscopic structure of scaffold plays a vital role to improve oxygen transfer [53, 54]. Figures 3(a) and 4(a) show relatively open interconnections in the microstructure of silk scaffolds before and after cell culture, respectively. Open pores on the surface of channel's wall generate an interface for proper oxygen diffusion.

Hollow channeled silk scaffolds, embodying 15 channels in two groups of SS2 (with channel diameter of 500 *μ*m) and SS3 (with channel diameter of 1000 *μ*m), alongside with unchanneled silk scaffold group (SS1), were specifically designed in order to investigate the effect of channel presence and also the consequence of its dimension on oxygen distribution across cell-seeded scaffolds. The pattern of channel distribution throughout the construct is a principal feature of our designed scaffold. There were different patterns that could be used, e.g., concentric circles, nested circles, parallel lines, or concentric diamonds, which could differ in channels' diameter, distance, and density throughout the construct. However, a privileged pattern that could contribute to a uniform oxygen distribution through scaffold would be more preferable. In our specific design, as depicted in Figure 1(b), channels are considered on the periphery of two hypothesized concentric circles with radius of 4 and 7 mm. It is apparent that channels of our design are distributed throughout the scaffold frame in an even manner; therefore, this compelling design enables an almost homogenous diffusion of oxygen and nutrients inside the scaffold and could prevent possible induced hypoxia in the depth of the constructs.

As shown in Figure 3(c), the fabricated natural silk scaffold made a network of interconnected pores sized from 100 to 300 *μ*m. The presented construct with average pore size of 200 *μ*m could result to an effective cell infiltration and migration. Moreover, Figure 3(c) exhibits an analogous porous structure of SS3 silk scaffold prior to cell seeding. The longitudinal cross-section of a channel in a SS2 scaffold at days 14 postseeding is demonstrated in Figure 4(a). The hollow channel presented in this micrograph shows that channel's wall still exhibits open pores two weeks after cell seeding and being placed in culture media which, on the other hand, contributes to a potential of oxygen availability in depth of the scaffold. Additionally, Figure 4(c) illustrates an attached cell's morphology on SS2 channeled silk fibroin scaffold. The attached and flattened G292 cells to the silk material have physiological osteoblastic morphologies.

Nevertheless, in the absence of channel, oxygen can diffuse into limited thickness of the porous structure adjacent to the surface (refer to Figures 5(c) and 8). Comparing Figures 5(a), 5(b), and 5(c), it could be claimed that the improvement of the macrostructure of the scaffold by insertion of channels results the elimination of oxygen limitation to a greater extent. Nine days postseeding, the oxygen level enhancement from ca. 0.6 ppm in unchanneled scaffolds to ca. 1.2 ppm in channeled scaffolds was found in our investigations (refer to Figure 5). The SS3 scaffold group demonstrates higher oxygen concentration at the center during the first week of cell culture compared to the SS2 group which is mainly due to more available surface area for oxygen molecules to diffuse into the interior spaces of SS3 scaffolds. Our records demonstrate that the effect of channel's diameter on oxygen transfer to the central areas of scaffold is remarkable although there is no linear relationship between channel's diameter and oxygen concentration at different points of scaffolds.

It can be observed from Figure 5(a) that the maximum difference between oxygen levels in the central points of scaffold groups was at 5^th^ day postseeding. Subsequently, the discrepancies between oxygen levels of all groups gradually diminished, where at day 9, the oxygen concentrations of the SS2 and SS3 groups approximately reached the same level. This phenomenon can be explained based on Alamar Blue assay results. According to Figure 7, metabolic activity of G292 cell seeded on SS3 scaffolds at the second week of culture exceeded far beyond SS2 scaffolds. Since, the higher metabolic activity needs more oxygen in the periphery milieu, G292 cell seeded on SS3 scaffolds consumed more oxygen compered to SS2 after the first days of culture. Therefore, as shown in Figure 5(a), the oxygen level at point A of SS3 sharps rapidly from day 5 to 9 leading to the shrinkage of oxygen level difference between the SS3 and SS2 groups. This phenomenon could be traced in Figure 5(b) too.

In addition to maintaining superior oxygen level and cell proliferation in the SS3 group, based on Figure 5, it could be concluded that the oxygen distribution through the center, middle, and surface points are approximately uniform for all days of culture. As presented by graphs in Figure 5, the oxygen concentration value at different points, for example on 7^th^ day, ranged between 2.5 and 3 ppm for SS3 and 1.5 and 3.8 ppm SS2 which corroborates the homogeneity of oxygen concentration in group SS3. Moreover, data showed that unchanneled scaffolds of SS1 did not provide uniform oxygen level through their structure; and therefore, hypoxia would be inevitable especially at the center because of poor accessibility to oxygen. As shown in Figure 5(a), oxygen concentration at the center of unchanneled scaffolds is much lower than oxygen concentration at the center of channeled scaffolds. Here, comparing the SS2 and SS3 groups shows how channel diameter could affect the oxygen transfer to the most remote area of the structure, i.e., the center.

Furthermore, Figure 5 interestingly makes evident that in the first week of cell culture, the rates of oxygen concentration reduction in all groups are higher than the second week. This phenomenon could be justified by the results presented in Figure 6 that demonstrates higher cell proliferation in the first week in comparison to the second week of culture. In addition, lower accessibility to oxygen for unchanneled scaffolds and the result of a lower cell growth in comparison with channeled constructs are recognizable in Figure 6.

**Figure 10:**
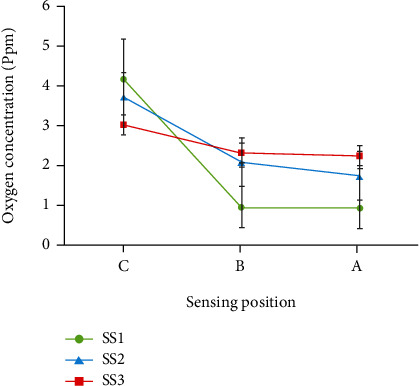
Oxygen concentration at day 7 for all groups of constructs at three points (A: at the scaffold center, B: at the distance of 680 *μ*m from the center, and C: at the scaffold surface).

In order to compare the oxygen level at the 3 points of sensing positions (shown in Figure 2), the oxygen concentrations of the SS1, SS2, and SS3 groups at all sensing points on day 7 of culture are depicted in Figure 10.

A remarkable outcome from Figure 10 is that the oxygen level difference between points B and C is greater than the difference between points A and B which could be due to the presence of diffusion barrier at the surface of the scaffold. Thus, more oxygen is available at the surface compared to other points inside the scaffolds.

In an analogous study, Rnjak-Kovacina et al. reported that introducing channels of diameter 570 *μ*m could improve oxygen level and cell distribution through the silk-based scaffold seeded by human dermal fibroblasts [43]. They used thinner scaffolds (with 1.2 mm diameter and 4 mm height), compared to the scaffolds designed and fabricated in this study.

In an effort done by Volkmer et al., it is shown that oxygen level would drop to 0% at the center of 3D unchanneled constructs seeded by osteoblasts after 5 days of culture and cells died due to induced hypoxia [5]. Likewise, we found that at the center of SS1 scaffolds, oxygen concentration diminished to 0.5 ppm after 9 days (Figure 5(a)). However, oxygen concentration values for channeled constructs were two or three times higher than that of unchanneled constructs at the same point and same day of culture.

In another study, a computational modeling predicted that the shape of channels could highly influence the oxygen distribution throughout 3D scaffolds [41]. Correspondingly, our investigation results demonstrated in Figure 10 indicate that increasing channel diameter could provide more homogenous distribution of oxygen throughout 3D sizeable silk scaffolds and elevates the oxygen availability to remote areas (i.e., point A at the center of scaffold).

## 5. Conclusion

Engineering of large tissue constructs needs special designs for scaffold structure to guarantee sufficient oxygen supply at its interior spaces and eliminate possible induced hypoxic regions in depth of the scaffolds. This work throws light on the effects of channeling on oxygen supply and deep-seated cell viability through large and clinically relevant sized tissue constructs. In conclusion, the straightforward and reproducible methodology introduced in this study could be utilized to fabricate 3D silk fibroin scaffolds with well-arranged and controllable hollow channels. Hollow channels are incorporated into the scaffold's structure to mimic the vascular networks in native tissues. The remarkable effect of channel diameter on oxygen supply to different areas of scaffold is well presented in this study. Therefore, the diameter of channels could optimize to yield enhanced oxygen delivery to the cells in the central areas of scaffold of any arbitrary design and eliminate possible induced hypoxia regions in depth. Moreover, the channel distribution pattern could be a notable parameter in modifying oxygen distribution pattern which should be investigated separately.

Furthermore, this work demonstrates that the design of SS3 scaffold with its specific channel configuration could appropriately supply oxygen and eliminate hypoxia regions in 3D sizeable silk scaffolds. Subsequently, such a design would enhance cell proliferation during two weeks of static culturing of human G292 osteoblast-like osteosarcoma cell line. Therefore, this channeled construct could potentially be served as a clinically relevant sized scaffold for a range of tissue engineering applications particularly for bone regeneration.

## Figures and Tables

**Table 1:** Characteristics of 3 silk scaffold prototypes.

Silk scaffold prototype	Diameter × height (cm)	Channeled	Channel diameter (*μ*m)
SS1	2.2 × 1	No	n.a.
SS2	2.2 × 1	Yes	500
SS3	2.2 × 1	Yes	1000

## Data Availability

Data is available upon request.
